# Safety and efficacy of CRS3123 in adults with a primary episode or first recurrence of *Clostridioides difficile* infection: a phase 2, randomised, double-blind, multicentre, vancomycin-controlled study

**DOI:** 10.1016/S1473-3099(25)00721-2

**Published:** 2026-01-22

**Authors:** Thomas Louie, Wendy Ribble, Louis Boccumini, Katherine Johnson, Mary A De Groote, Joshua Day, Clifford Mason, Xicheng Sun, Jane Freeman, Kenan Gu, Glenn Tillotson, Mark H Wilcox, Nebojsa Janjic, Thale C Jarvis, Seema U Nayak, Urs A Ochsner, Jon B Bruss

**Affiliations:** Foothills Medical Center, Calgary, AB, Canada; Crestone, Boulder, CO, USA; Crestone, Boulder, CO, USA; Crestone, Boulder, CO, USA; Crestone, Boulder, CO, USA; Crestone, Boulder, CO, USA; Crestone, Boulder, CO, USA; Crestone, Boulder, CO, USA; University of Leeds, Leeds, UK; National Institute of Allergy and Infectious Diseases, National Institutes of Health, Bethesda, MD, USA; GST Micro LLC, North, VA, USA; University of Leeds, Leeds, UK; Crestone, Boulder, CO, USA; Crestone, Boulder, CO, USA; National Institute of Allergy and Infectious Diseases, National Institutes of Health, Bethesda, MD, USA; Crestone, Boulder, CO, USA; Crestone, Boulder, CO, USA

## Abstract

**Background:**

CRS3123 is a potent inhibitor of protein synthesis in bacteria that express type 1 methionyl-tRNA ligase, resulting in selective antibacterial activity that holds promise as a novel treatment for *Clostridioides difficile* infection (CDI). The purpose of this study was to evaluate the safety and efficacy of CRS3123 in adults with a primary episode or first recurrence of CDI.

**Methods:**

This multicentre, randomised, double-blind, vancomycin-controlled, phase 2 study was conducted across 14 enrolling sites in the USA and Canada. Enrolled patients were 18 years or older, with a clinical diagnosis of CDI, including diarrhoea (at least three unformed bowel movements in the 24 h before randomisation) and *C difficile* toxin A or B, or both, detected in stools. Patients were excluded if they had more than one episode of CDI within the past 3 months or more than two within the past 12 months. Patients were randomly assigned (1:1:1) to receive 10 days of treatment with one of two dose regimens of CRS3123 (200 mg and 400 mg orally twice daily) or oral vancomycin (125 mg orally four times daily). Randomisation used an interactive response technology system and was stratified by history of CDI (first episode *vs* first recurrence within the past 3–12 months). Masking was maintained by use of matching dummy capsules. Safety was a primary endpoint and was assessed in patients who received at least one dose of study drug. The primary efficacy endpoint was clinical cure (survival and resolution of diarrhoea) at the test-of-cure (TOC) visit (day 12 up to day 15) in the intention-to-treat (ITT) population. The rate of CDI recurrence was a crucial secondary outcome measure assessed at study days 40 and 70 in the microbiological ITT population, which included all patients in the ITT population who tested positively for *C difficile* toxin at screening or day 1 and who had *C difficile* isolated in culture. This trial was registered with ClinicalTrials.gov (NCT04781387) and is complete.

**Findings:**

Between May 12, 2021, and April 26, 2024, 58 individuals were assessed for eligibility, 43 of whom were recruited and randomly assigned: 14 (33%) to the CRS3123 200 mg group, 15 (35%) to the CRS3123 400 mg group, and 14 (33%) to the vancomycin group. The mean age of patients was 58·4 years (SD 18·0), 33 (77%) patients were female, and ten (23%) patients were male. 31 (72%) patients had a primary episode of CDI and 12 (28%) patients had a first recurrence of CDI. All patients received at least one dose of the assigned drug. Treatment-emergent adverse events (TEAEs) were mild to moderate in severity and similar across treatment groups. TEAEs considered possibly related to study drug (all grade 1 or 2) were reported in one patient in the CRS3123 200 mg group (dry mouth, asthenia and gastro-oesophageal reflux disease, nausea, vomiting, increased alanine aminotransferase, and increased aspartate aminotransferase), three patients in the CRS3123 400 mg group (one with increases in alanine aminotransferase, aspartate aminotransferase, and alkaline phosphatase; one with malaise, nausea, and feeling abnormal; and one with headache); and in no patients in the vancomycin group. The only serious adverse event reported, pneumonia, was in the vancomycin group and was considered unrelated to the study drug. 13 (93%) of 14 patients in the CRS3123 200 mg group, all 15 (100%) patients in the CRS3123 400 mg group, and 13 (93%) of 14 patients in the vancomycin group had clinical cure at the TOC visit. No patient had clinical failure at the TOC visit; two patients (one each in the CRS3123 200 mg and vancomycin groups) had indeterminate responses due to missing data. Recurrence through day 40 occurred in one (7%) of 14 patients in the CRS3123 400 mg group, none of the 13 patients in the CRS3123 200 mg group, and three (23%) of 13 patients in the vancomycin group, and there was an additional recurrence on day 70 in the CRS3123 400 mg group.

**Interpretation:**

Both doses of CRS3123 were deemed safe and well tolerated and showed efficacy similar to vancomycin at the TOC visit, with lower rates of recurrence. Together, these data support further development of CRS3123.

**Funding:**

US National Institute of Allergy and Infectious Diseases at the National Institutes of Health, Department of Health and Human Services.

## Introduction

*Clostridioides difficile* infection (CDI) remains a global health threat in both hospital and community settings.^1^ *C difficile* exotoxins A and B are primarily responsible for severe diarrhoea and pseudomembranous colitis—the most common manifestations of CDI. CDI is a condition that does not tend to resolve spontaneously.^2^ CDI mortality rates vary widely; however, 30-day mortality rates are reported to be as high as 6–13% (similar to rates seen in sepsis, meningococcal infection, and other similar conditions) and can be higher in older patients with clinically relevant comorbidities.^3,4^

Healthy intestinal microbiota, which serves as a main barrier to gut colonisation by ubiquitous environmental pathogens such as *C difficile*, is often disrupted by broad-spectrum antibiotic therapy, leading to dysbiosis and increased risk of CDI. Persistent disruption of normal microbiota during treatment of CDI is a driving factor in the recurrence of CDI,^5,6^ which remains a major clinical challenge with the currently available treatment options.^7,8^ Recurrence rates often exceed 20% after treatment of an initial episode with vancomycin, one of the most widely used therapies for CDI.^9–13^ For this reason, the somewhat narrower-spectrum agent fidaxomicin has become a preferred treatment for CDI in recent guidelines.^14^ Alternative treatments that can be effective in the acute infection stage and preserve the normal gut microbiota to reduce the risk of recurrence are needed.

CRS3123 has emerged as a promising new drug candidate for CDI in large part due to its exceptionally narrow spectrum coupled with high potency against *C difficile*.^15,16^ The narrow spectrum of CRS3123 is a direct consequence of the phylogenetic distribution of its molecular target, methionyl-tRNA synthetase type 1 (MetRS1), now also known as methionyl-tRNA ligase type 1 (UniProt Q181D9), which is found in most aerobic Gram-positive bacteria, including many known pathogens, and in some Gram-positive anaerobes like *C difficile*. Most Gram-negative bacteria and many commensal anaerobes rely on a distinct type 2 enzyme (methionyl-tRNA ligase type 2 [UniProt POO989]) against which CRS3123 is completely inactive.^15,17^ CRS3123 potently inhibits MetRS1 by competitively displacing methionine from the active site, thus preventing tRNA charging and subsequent protein synthesis, leading to rapid inhibition of toxin production and sporulation.^18^

In pre-clinical and phase 1 clinical testing, CRS3123 showed several other desirable characteristics for a CDI drug candidate, including low oral systemic bioavailability, high intestinal concentrations, minimal disruption of commensal microbiota, and a favourable safety and tolerability profile.^16,19^ We advanced CRS3123 into phase 2 testing to evaluate its safety and efficacy in patients with CDI. We enrolled only patients with toxin-positive CDI based on EIA or ELISA testing to differentiate CDI from other conditions with similar clinical symptoms.^20,21^ We chose vancomycin as a comparator because of its frequent clinical use, as well as its established rates of initial cure and recurrence in numerous clinical trials, which provided a benchmark against which CRS3123 could be evaluated.^14,22,23^

## Methods

### Study design

We did a phase 2, randomised, multicentre, double-blind, comparator-controlled clinical trial of two dose levels of orally administered CRS3123 and vancomycin in patients with toxin-confirmed CDI in the USA and Canada. The study was done between May 12, 2021, and April 26, 2024, at 14 outpatient clinics and hospital-associated sites (two in Canada and 12 in the USA). The study was registered with ClinicalTrials.gov on Jan 5, 2021 (NCT04781387) and is complete. The protocol, informed consent form, and other appropriate materials were approved by Advarra Central Institutional Review Board (IRB; registration number 00000971) before implementation. An independent Data and Safety Monitoring Board (DSMB) was organised and managed by the US National Institute of Allergy and Infectious Diseases (NIAID). These materials and the statistical analysis plan (SAP) can be accessed at ClinicalTrials.gov.

### Patients

Enrolled patients were 18 years or older with a clinical diagnosis of CDI, including diarrhoea (at least three unformed bowel movements [UBMs] in the 24 h before randomisation) assessed as Bristol Stool Scale score 5, 6, or 7 and that was judged by an investigator to be caused by *C difficile*, and the presence of *C difficile* toxin A or B, or both, in stools as detected by an EIA or ELISA laboratory test approved and cleared by the US Food and Drug Administration (FDA) or Health Canada. Written informed consent was obtained from all patients, using the Advarra IRB approved form.

The qualifying stool sample must have been produced, and other screening procedures completed, within 72 h before randomisation. The CDI episode at enrolment had to be either a first episode or a first recurrence. Patients were excluded if they had more than one episode of CDI within the past 3 months or more than two CDI episodes within the past 12 months before randomisation. Patients were also excluded if they had received more than a 24-h course of standard-of-care CDI antibiotic treatment before randomisation. Patients with fulminant CDI, toxic megacolon, septic shock, intestinal ileus, colonic perforation, underlying inflammatory bowel disease (Crohn’s disease or ulcerative colitis), short bowel syndrome, untreated Hirschsprung’s disease, or any other gastrointestinal condition that might affect gastrointestinal motility or absorption from the gastrointestinal tract were excluded. A complete list of eligibility criteria and a summary of protocol deviations are provided in the appendix (p 5).

### Randomisation and masking

Patients were randomly assigned in a 1:1:1 ratio to one of three orally administered treatment regimens using balanced block randomisation, with block sizes of three or six, within each stratification group. Patients were stratified by history of CDI (first episode or first recurrence within the past 3–12 months) and were assigned to CRS3123 200 mg twice daily, CRS3123 400 mg twice daily, or vancomycin 125 mg four times daily. Patients received study medication for 10 days. Patients recorded the date and time of each dose on a paper diary that was reviewed at each study visit and collected after all doses had been taken. An interactive response technology system—a module of the electronic data capture software (iMedNet, Minnetonka, MN, USA)—was used to assign an appropriate blister pack of study treatment based on the randomisation, which was provided to the masked study site’s pharmacist or designee for self-administration of the study drug by the patient. Masking was ensured by using identical size 0, light blue, opaque, hard-gelatin capsules for the study drug (CRS3123), the comparator (over-encapsulated vancomycin), and dummy capsules containing inert ingredients only, so that all products matched in appearance and the differences in dose strength and dosing intervals were concealed. The allocations were concealed in the interactive response technology system to avoid selection bias. The masking was maintained throughout the trial until the database was locked. All patients, investigators, pharmacists, outcome assessors, personnel, and those involved in all clinical evaluations were masked to treatment assignment throughout the study. The study sponsor and clinical research organisation were also masked to treatment assignment.

### Procedures

All participants took the study treatment four times daily at approximately 6-h intervals for a total of four doses per day, for a total of 10 days. CRS3123 (200 mg and 400 mg, respectively) was given twice a day orally as doses one and three, with matching dummy capsules given as doses two and four. The vancomycin comparator was given as 125 mg capsules orally four times a day. Each patient attended clinic visits at prespecified timepoints during the study for the evaluation of safety, efficacy, and pharmacokinetics. Key visits included enrolment and randomisation (day 1), the end-of-treatment (EOT) visit (day 10 up to day 12), the test-of-cure (TOC) visit for clinical response (day 12 up to day 15), the long-term follow-up visit for sustained clinical response (day 40 or day 37 up to day 43), and assessment of the continuation of cure (day 70 or day 67 up to day 73). The study investigator or subinvestigator at each site ensured these timeframes were followed. Bowel movement number and Bristol Stool Scale scores were reported on patient diaries and discussed with investigators at study visits. Plasma was collected for pharmacokinetic analysis on day 1 and at the EOT visit. Stool was collected for *C difficile* culture, *C difficile* spore quantitation (JMI Laboratories, North Liberty, IA, USA), and *C difficile* semi-quantitative toxin testing and ribotyping (University of Leeds and UKHSA *C difficile* Ribotyping Network Laboratory, Leeds, UK), as described in the appendix (p 2). Safety was assessed by adverse events reported in patient diaries and discussed at visits, physical examinations, vital signs, electrocardiogram (ECG) parameters, and laboratory evaluations (haematology, chemistry, and urinalysis) on day 1, day 4, EOT, TOC or follow-up visit 1, and follow-up visit 2 (day 40, 30 days after the last day of therapy) visits. The Common Terminology Criteria for Adverse Events version 5.0 was used for grading scales of adverse events and the principal investigator was responsible for judging relatedness. Coagulation was assessed at screening and EOT visits only and complete blood count with differential at the TOC visit. Quality of life parameters were assessed from screening until the TOC visit, and at episodes of CDI recurrence if applicable, using the *Clostridium difficile* Infection–Daily Symptoms patient-reported outcome (CDI–DaySyms PRO) questionnaire.^24,25^ The CDI-DaySyms PRO questionnaire was filled out in paper format daily by the patient through the TOC visit only. Sex data were collected on the case report form, which had two options (male or female) and was filled out by the investigator.

### Outcomes

Safety data were reviewed by designated medical monitors at Crestone (Boulder, CO, USA), the contract research organisation, the NIAID’s clinical team, and by the DSMB, with regular reports submitted to the NIAID. Efficacy endpoints were reviewed by sponsor representatives to ensure compliance with the study protocol.

Safety and tolerability of CRS3123 was a primary outcome, based on: physical examinations, vital signs, laboratory evaluations (haematology, blood chemistry, coagulation, and urinalysis), ECGs, and treatment-emergent adverse events (TEAEs).

The primary efficacy endpoint was the rate of clinical cure at the TOC visit in the intention-to-treat (ITT) population. Clinical cure was defined as survival through the TOC visit and resolution of diarrhoea. Resolution of diarrhoea was defined as fewer than three UBMs per day at the EOT visit, with maintenance of resolution through the TOC visit and no further requirement (in the investigator’s judgement) for CDI treatment through the TOC visit. Patients who did not show clinical cure were either classified as having clinical failure or an indeterminate response. Clinical failure was defined as persistence or return of diarrhoea (at least three UBMs per day), the need for additional (non-study) anti-CDI treatment through the TOC visit, or mortality before the TOC visit. An indeterminate response was defined as patients for whom study data were unavailable for evaluation of clinical response at the TOC visit for any reason, or who were classified as clinical failure at the TOC visit but took medication that can alter normal gastrointestinal flora (eg, systemic antibiotics) or received therapy that can cause diarrhoea (eg, laxatives), or who were classified as clinical cure at the TOC visit but took medication that can cause constipation or decrease in stool frequency (eg, loperamide). Concomitant medications were monitored and documented by the principal investigator and added to the diary by the patient.

Key secondary outcomes included rate of clinical cure at the TOC visit as assessed by the investigator in the microbiological ITT (micro-ITT), per-protocol, and microbiologically evaluable populations; rate of recurrence of CDI through day 40 and day 70, and time to recurrence of CDI up to day 70, in the micro-ITT and microbiologically evaluable populations; and rate of sustained clinical response in the micro-ITT, per-protocol, and microbiologically evaluable populations. Recurrent CDI was defined as a new episode of diarrhoea (at least three UBMs per day) with a positive test for *C difficile* toxin (QUIK CHEK COMPLETE stool test provided by TechLabs [Blacksburg, VA, USA] or other another EIA or ELISA toxin test approved and cleared by the FDA or Health Canada). Sustained clinical response was defined as clinical response at the TOC visit and no recurrence of CDI through study day 40 (30 days after EOT visit).

Other key secondary outcomes were rates of total relief of symptoms and time to resolution of diarrhoea measured through the TOC visit in the micro-ITT, per-protocol, and microbiologically evaluable populations; pharmacokinetics until the EOT visit in the pharmacokinetic population (any patient that received at least one dose of CRS3123; see full pharmacokinetic sampling schedule in study protocol); and the CDI–DaySyms PRO questionnaire symptom score assessed in the ITT population daily from baseline through the TOC visit and at suspected recurrence. Total relief of symptoms was defined at the TOC visit as resolution (less than three per day) of UBMs (ie, return to Bristol Stool Scale scores 1–4 recorded on the participant daily diary), an investigator’s assessment of clinical cure, and the resolution of signs or symptoms of CDI recorded at baseline (ie, fever [>37·7 °C], abdominal pain [as assessed by the investigator], and white blood cells higher than 15 000 cells per μL). Prespecified exploratory outcomes reported in this study were positive *C difficile* culture (for inclusion in the micro-ITT and microbiologically evaluable populations), *C difficile* spore quantitation (spore counts over time and change from baseline to the TOC visit), and *C difficile* semi-quantitative toxin testing (toxin titres over time and change from baseline to the TOC visit) and ribotyping (proportions with hypervirulent ribotypes), all assessed in the micro-ITT population. Additional exploratory outcomes, to be reported separately, include microbiome, metabolome, and inflammation biomarker analysis.

### Statistical analysis

Full statistical methods are described in detail in the SAP, including how missing data were handled. The target enrolment (90–108 patients) was not powered for hypothesis testing but was chosen to provide a reasonable estimate of the primary endpoint to inform future trials. Actual enrolment was lower (n=43), primarily due to the COVID-19 pandemic (appendix p 3).

Safety was assessed in the safety population, which included all patients who received any amount of study treatment and were analysed by the treatment received. The per-protocol population included all randomly assigned patients who received at least 80% of the study treatment; received no concomitant systemic antibiotics through the TOC visit; received no prohibited probiotic or anti-diarrhoeal medication through the TOC visit; and had an investigator-assessed clinical response at the TOC visit and no major protocol deviations. The ITT population included all randomly assigned patients. The micro-ITT population included all patients in the ITT population who tested positively for *C difficile* toxin at screening or day 1 and who had *C difficile* isolated in culture from a sample taken at baseline. The microbiologically evaluable population included all patients in the per-protocol population who tested positively for *C difficile* toxin at screening or day 1 and who had *C difficile* isolated in culture from a sample taken at baseline. A schematic of study populations and definitions is provided in the appendix (p 4).

All analyses were conducted using SAS version 9.4 or higher. Continuous variables were summarised using absolute numbers and percentages, means and SDs, medians and IQRs, and minimum and maximum values. Summaries of pharmacokinetic plasma concentrations also include the geometric mean and coefficient of variation. Exact confidence intervals around proportions were derived with the Clopper–Pearson method. Confidence intervals around differences in proportions between CRS3123 groups and the vancomycin group were obtained with the Miettinen and Nurminen method. The time to resolution of diarrhoea was defined as the time elapsed in (decimal) days from randomisation to the last UBM before 2 consecutive days of fewer than three UBMs (Bristol Stool Scale score of 5, 6, or 7) per day through the TOC visit. If a participant never had resolution of diarrhoea through the TOC visit, then the time to resolution of diarrhoea was censored as the time of the last bowel movement on or before the TOC visit. When the event did not occur as reported by the patient, the last date and time the participant reported no resolution (ie, at least three UBMs) was used as the censored time measurement. The date and time of reporting no resolution coincided with the last reported UBM date and time in all cases. The investigator assessment form and the participant daily diary form, which were filled out daily from screening to the TOC visit, were used to assess the last available date without resolution through the TOC visit. Median time to recurrence (five events overall) could not be evaluated in any treatment group using Kaplan–Meier methods due to the low number of recurrences.

### Role of the funding source

The funder of the study (US National Institute of Allergy and Infectious Diseases at the National Institutes of Health, Department of Health and Human Services), including authors SUN and KG, provided collaborative subject matter expertise in the study design, data collection, data analysis, data interpretation, and review of the Clinical Study Report (final study document signed by the sponsor and clinical research organisation that contains the synopsis and all study data, is linked to all non-text items, and is reviewed by the US FDA), but were not involved in writing.

## Results

**Figure 1:**
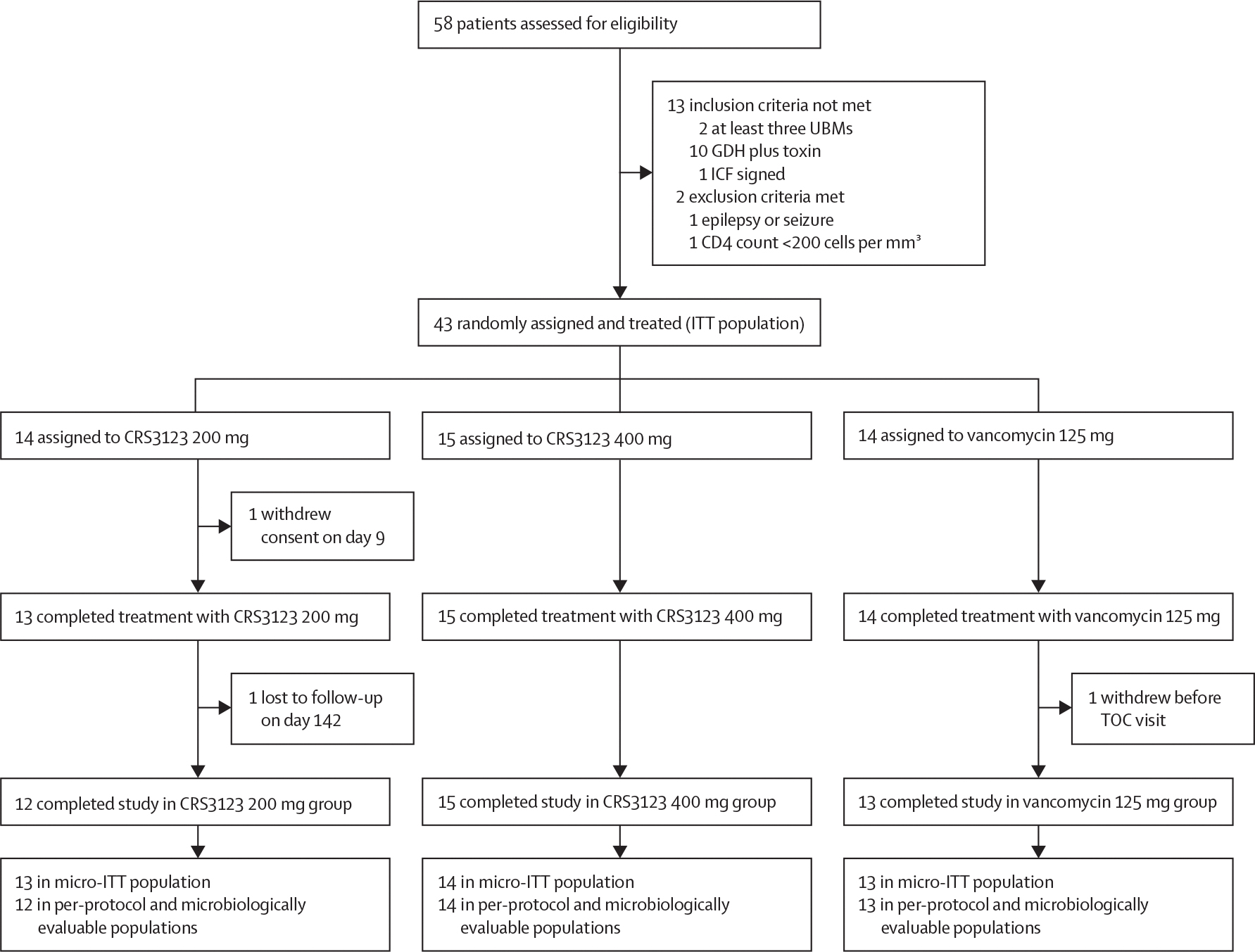
Patient disposition and populations In the micro-ITT population, three patients included in the ITT population were excluded, one each in the CRS3123 200 mg and 400 mg groups because they did not have a positive toxin A or B test, and one in the vancomycin group because they did not have a positive *C difficile* culture. In the per-protocol and microbiologically evaluable populations, which were identical, one additional patient in the CRS3123 200 mg group was excluded because the patient did not receive at least 32 doses of the study drug (withdrew consent on day 9). *C difficile*=*Clostridioides difficile*. GDH plus toxin=positive enzyme immunoassay for glutamate dehydrogenase and toxin A or B. ICF=informed consent form. ITT=intention-to-treat. Micro-ITT=microbiological ITT. TOC=test-of-cure. UBMs=unformed bowel movements.

58 patients were screened for eligibility and 43 patients were randomly assigned and treated in the study between May 12, 2021, and April 26, 2024, including 14 patients in both the CRS3123 200 mg and vancomycin 125 mg groups, and 15 patients in the CRS3123 400 mg group (figure 1). These patients made up the ITT and safety populations. The micro-ITT population included 13 patients in both the CRS3123 200 mg and vancomycin groups and 14 patients in the CRS3123 400 mg group; two patients were excluded, one each in the CRS3123 200 mg and 400 mg groups, because they did not have a positive toxin A or B test, and one patient in the vancomycin group was excluded because they did not have a positive *C difficile* culture. As well as these patients, one patient in the CRS3123 200 mg group was excluded from the per-protocol and microbiologically evaluable populations, which were identical, due to withdrawing on day 9 of the study after receiving less than 32 doses of study treatment. 40 (93%) of 43 patients in the ITT population completed the study. Three patients, including the patient who withdrew on day 9, did not complete the study (figure 1; appendix p 4). Protocol deviations are listed in the appendix (p 7).

Baseline demographic and disease characteristics were generally similar between treatment groups, with a few notable differences (table 1). The vancomycin treatment group had a higher mean age of 65·0 years (SD 15·1) compared with 55·2 years (18·6) for the combined CRS3123 treatment groups. This difference was due to one older patient (ie, age 90 years) in the vancomycin group and one younger patient (ie, age 20 years) in the combined CRS3123 groups. Baseline characteristics were assessed for each of the three treatment groups and the CRS3123 groups combined to support the other combined analyses (SAP sections 8.6 and 9.6.3). More participants were 65 years or older in the vancomycin group than in the CRS3123 groups (50% *vs* 29% and 33% in the CRS3123 400 mg and 200 mg groups, respectively). The combined CRS3123 groups had a higher mean BMI of 28·0 kg/m^2^ (SD 6·5), compared with a mean BMI of 25·4 kg/m^2^ (5·5) in the vancomycin group. There were more male participants in the vancomycin group than in the combined CRS3123 groups (29% *vs* 14% and 27% in the CRS3123 200 mg and 400 mg groups, respectively). There was a difference in the proportion of participants with a white blood cell count higher than 15 000 cells per μL (14% in the CRS3123 200 mg and vancomycin groups and 0% in the CRS3123 400 mg group).

28 (65%) of the 43 patients in the safety population had at least one TEAE (table 2). All but one of the TEAEs were mild to moderate (grade 1 or 2) in severity. The most frequent TEAEs were diarrhoea, abdominal pain, headache, and nausea. Diarrhoea and abdominal pain were reported more frequently in the vancomycin group than in either CRS3123 group or in the combined CRS3123 groups. Headache and nausea were only reported in the CRS3123 groups. Additional TEAEs in two or more patients were vomiting, muscle spasms, rash, and abdominal distension. Two patients in the CRS3123 400 mg dose group reported a grade 1 rash that was considered unrelated to study treatment. One patient’s rash started from day 30 and resolved on day 45, whereas the other patient’s rash started on day 7 and resolved on day 10. There was a single grade 3 TEAE (recurrence of diarrhoea in a patient in the vancomycin group), which was deemed unrelated to the study drug. Two adverse events (grade 2 asthenia and grade 1 gastro-oesophageal reflux disease), both considered possibly related to study drug, led to early treatment discontinuation in one patient in the CRS3123 200 mg group, who also had dry mouth, nausea, vomiting, increased alanine aminotransferase, and increased aspartate aminotransferase. Three other patients reported TEAEs that were considered possibly related to the study drug by the investigator. All three patients were in the CRS3123 400 mg group, including one patient who reported several TEAEs of feeling abnormal, general malaise, and nausea; one patient with headache; and one patient with increased alanine aminotransferase, aspartate aminotransferase, and alkaline phosphatase. One patient in the vancomycin group with a history of chronic obstructive pulmonary disease had a serious adverse event (SAE) of pneumonia requiring hospitalisation. This SAE was grade 2 and considered unrelated to the study drug. No participant who received CRS3123 had an SAE.

Changes in laboratory parameters, vital signs, and ECGs were generally small and not considered clinically significant (data not shown). Of 37 patients with available ECG data (ie, PR interval, QRS duration, QT interval, and RR interval), 17 (46%; including five [50%] of ten patients in the CRS3123 200 mg group, four [27%] of 15 patients in the CRS3123 400 mg group, and eight [67%] of 12 patients in the vancomycin group) showed a QT interval corrected using Fridericia’s formula increase of more than 0 ms to less than 30 ms compared with baseline. A single patient in the CRS3123 400 mg group showed a QTcF increase of at least 30 ms to less than 60 ms, with values of 408 ms at baseline and 446 ms on day 10 (ie, EOT visit). The overall ECG interpretation was normal for this one patient.

High clinical cure rates at the TOC visit were observed in all three treatment groups in the ITT population—specifically, in 13 (93%) of 14 patients in the CRS3123 200 mg group, in 15 (100%) of 15 patients in the CRS3123 400 mg group, and in 13 (93%) of 14 patients in the vancomycin group (table 3). No patient had clinical failure at the TOC visit. There were two indeterminate responses (one in the CRS3123 200 mg group and one in the vancomycin group). One patient treated with CRS3123 200 mg withdrew on day 9 of the study before completing treatment, and one patient treated with vancomycin completed treatment but withdrew on day 11 before the TOC visit. Both patients appeared to be successfully treated as of last contact; however, they were included in the ITT population and statistically count as failure (ie, not having clinical cure).

Clinical cure rates at the TOC visit in key secondary analysis populations were consistent with clinical cure rates in the ITT population. In the micro-ITT population, 12 (92%) of 13 patients in the CRS3123 200 mg group, 14 (100%) of 14 patients in the CRS3123 400 mg group, and 13 (100%) of 13 patients in the vancomycin group had clinical cure at the TOC visit. One indeterminate response was observed in the CRS3123 200 mg group because the participant withdrew from the study on day 9 and therefore data for clinical cure at the TOC visit were unavailable. The one patient in the vancomycin group in the ITT population with an indeterminate response was excluded from the micro-ITT population because they did not have a positive *C difficile* culture result. Clinical cure at the TOC visit in the per-protocol and microbiologically evaluable populations was identical at 12 (100%) of 12 patients in the CRS3123 200 mg group, 14 (100%) of 14 patients in the CRS3123 400 mg group, and 13 (100%) of 13 patients in the vancomycin group (table 3).

Rates of clinical cure at the TOC visit in the ITT population were similar across stratification subgroups. In randomly assigned patients with a primary episode of CDI (n=32), ten (91% [95% CI 59–100]) of 11 patients in the CRS3123 200 mg group), 11 (100% [72–100]) of 11 patients in the CRS3123 400 mg group, and ten (100% [69–100]) of ten patients in the vancomycin 125 mg group had clinical cure at the TOC visit. In randomly assigned patients with a first recurrence of CDI (n=11), three (100% [29–100]) of three patients in the CRS3123 200 mg group, four (100% [40–100]) of four patients in the CRS3123 400 mg group, and three (75% [19–99]) of four patients in the vancomycin group had clinical cure at the TOC visit. It is worth noting that among patients with a first recurrence, there was wide variation (from 16 days before to 3144 days before randomisation) in the reported date of previous CDI episode.

**Figure 2:**
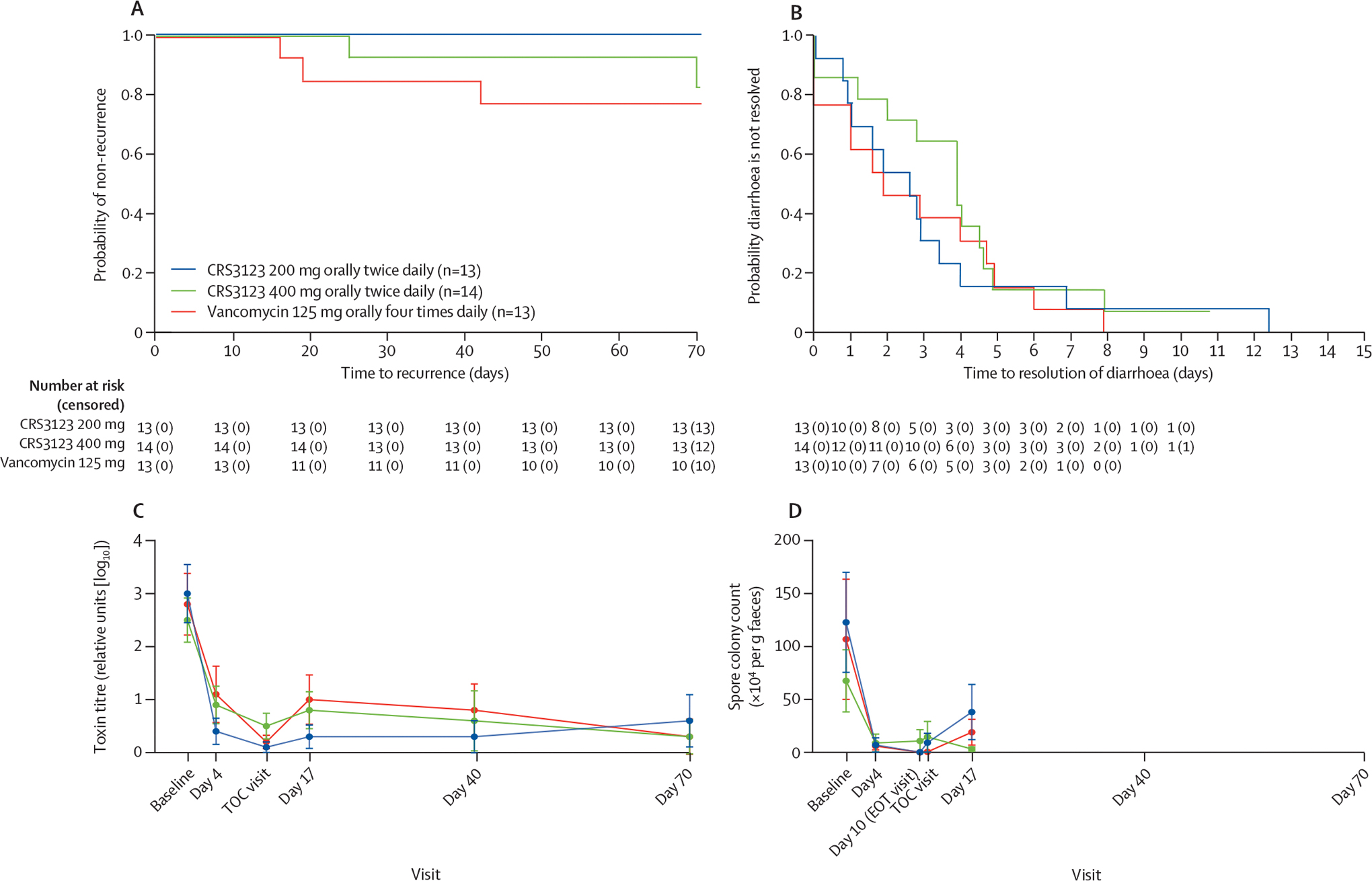
Clinical and laboratory parameters over time in the three treatment groups of the micro-ITT population (A) Kaplan–Meier curves for recurrence through day 70, shown by treatment group. Patients were censored if they never had recurrence or had missing data. Censor time was the last visit in which recurrence did not occur. (B) Kaplan–Meier curves for diarrhoea (at least three UBMs in 24 h assessed as Bristol Stool Scale scores ≥5). Patients were censored if they never had resolution of diarrhoea at the time of their last bowel movement before the TOC visit. (C) Mean *C difficile* toxin A and B titres, as assessed by the CCNA. The figure excludes toxin titres collected on or after the date of recurrence. (D) Viable spore colony count, as assessed via culture following ethanol-shock killing of vegetative *C difficile* cells. The figure excludes spore counts collected on or after the date of recurrence and spores were undetectable in all but one participant beyond day 17. CCNA=cell cytotoxicity neutralisation assay. *C difficile*=*Clostridioides difficile*. EOT=end-of-treatment. Micro-ITT=microbiological intention-to-treat. TOC=test-of-cure. UBM=unformed bowel movement.

In the micro-ITT population, 12 (92%) of 13 patients in the CRS3123 200 mg group, 13 (93%) of 14 patients in the CRS3123 400 mg group, and ten (77%) of 13 patients in the vancomycin group had a sustained clinical response (table 4). In the micro-ITT population, there were no early recurrences (between the TOC visit and day 40) of CDI in the CRS3123 200 mg group. In the CRS3123 400 mg group, one (7%) of 14 patients had an early recurrence on day 25, and in the vancomycin group, three (23%) of 13 patients had an early recurrence on days 16, 19, and 42 (figure 2A). Participants with recurrence were considered as having clinical cure at the TOC visit with diarrhoea resolved but had a recurrence of diarrhoea with a positive toxin test during follow-up. Rates of sustained clinical response in the per-protocol and microbiologically evaluable populations were similar to those in the micro-ITT population, except for the CRS3123 200 mg group, from which the patient with an indeterminate response at the TOC visit was excluded, and therefore the sustained clinical response rate was 100% (12 of 12 patients) in that group (table 4). In an analysis of the rate of recurrence of CDI through day 70 in the micro-ITT and microbiologically evaluable populations, there were no recurrences of CDI through day 70 in the CRS3123 200 mg group, two recurrences in the CRS3123 400 mg dose group (one on day 25 and one on day 70), and three recurrences in the vancomycin group (table 4; figure 2A). The rates of recurrence through day 70 in the micro-ITT population were 0% (95% CI 0–26) in the CRS3123 200 mg group, 14% (2–43) in the CRS3123 400 mg group, and 23% (5–54) in the vancomycin group.

Diarrhoea resolved in most patients by day 5, associated with a decline in toxin A and B titres, with some residual diarrhoea continuing in a few patients (figure 2B). Median time to resolution of diarrhoea in the micro-ITT population was 2·6 days (95% CI 0·9–3·4) in the CRS3123 200 mg group, 3·9 days (1·2–4·6) in the CRS3123 400 mg group, and 1·9 days (0–4·7) in the vancomycin group. The persistence of some UBMs, reported by a few patients between the EOT and TOC visits, was not associated with any of the instances of recurrence that occurred later in follow-up (ie, they showed clinical cure at the TOC visit and did not have recurrence at day 40 or day 70 visits). In the micro-ITT population, total relief of symptoms at the TOC visit was reported by 11 (85% [95% CI 55–98]) patients in the CRS3123 200 mg group, 14 (100% [77–100]) patients in the CRS3123 400 mg group, and 12 (92% [64–100]) patients in the vancomycin group. Treatment differences versus the vancomycin group in rates of total relief of symptoms were –8 (95% CI –37 to 22) and 8 (–15 to 34) percentage points for the 200 mg and 400 mg groups, respectively. In the per-protocol and microbiologically evaluable populations, total relief of symptoms at the TOC visit was identical and reported by 11 (92% [95% CI 62–100]) patients in the CRS3123 200 mg group, 14 (100% [77–100]) patients in the CRS3123 400 mg group, and 12 (92% [64–100]) patients in the vancomycin group. Analysis of the CDI–DaySyms PRO questionnaire results showed no clinically relevant differences between treatment groups (data not shown). Mean diarrhoea symptoms, abdominal symptoms, and systemic and other symptoms domains were similar across treatment groups at baseline, and each domain score decreased steadily over time, with no notable differences between treatment groups.

*C difficile* toxin titres decreased rapidly over time in all three treatment groups (figure 2C). Mean toxin titres at baseline in the micro-ITT population were 3·0 (SD 1·9) relative (log_10_) units in the CRS3123 200 mg group, 2·5 (1·5) relative units in the CRS3123 400 mg group, and 2·8 (2·1) relative units in the vancomycin group. By the TOC visit, mean toxin titres were 0·1 (SD 0·3) relative units in the CRS3123 200 mg group, 0·5 (0·9) relative units in the CRS3123 400 mg group, and 0·2 (0·4) relative units in the vancomycin group, corresponding to changes from baseline of –2·8 (SD 2·1), –2·0 (1·7), and –2·8 (2·4) relative units, respectively.

Similarly, spore counts rapidly declined in all groups. Mean spore colony count × 10^4^ colony forming units per g of faeces at baseline in the micro-ITT population was 123 (SD 170) in the CRS3123 200 mg group, 68 (110) in the CRS3123 400 mg group, and 107 (197) in the vancomycin group. By the TOC visit, mean spore colony count × 10^4^ colony forming units per g of faeces was 9 (SD 30) in the CRS3123 200 mg group, 15 (53) in the CRS3123 400 mg group, and 0·43 (1·5) in the vancomycin group, corresponding to mean changes from baseline of –98 (SD 179), –58 (71), and –106 (196), respectively. Figure 2D illustrates the change to day 17, excluding data collected at or after recurrence. Spores were undetectable in all but one participant in the CRS3123 200 mg treatment group beyond day 17.

Hypervirulent *C difficile* strain ribotype 027 was isolated in one patient in the 200 mg CRS3123 group and two patients in the vancomycin group at baseline. Hypervirulent strain ribotype 078 was found in two patients in the vancomycin group at baseline. All three patients with ribotype 027 isolated at baseline showed clinical cure at the TOC visit, with no CDI recurrences through day 70. Both participants with ribotype 078 isolated at baseline showed clinical cure at the TOC visit, but were reported as early recurrences through day 40, and in one of them, the strain causing the recurrence was also ribotype 078.

The plasma and faecal pharmacokinetic population comprised 29 participants: 14 (48%) in the CRS3123 200 mg group and 15 (52%) in the CRS3123 400 mg group. Consistent with phase 1 studies,^16^ there were substantial faecal concentrations—2–3 orders of magnitude above CRS3123 minimum inhibitory concentration values for *C difficile* throughout the treatment period—with relatively lower (approximately 1000-fold) levels detected in plasma. Mean pre-dose faecal concentrations at the EOT visit were 1210 (SD 1210) μg/g in the CRS3123 200 mg group and 2920 (1880) μg/g in the CRS3123 400 mg group, indicating that CRS3123 largely remained in the gastrointestinal tract. At 7 days after the last dose, one (9%) of 11 participants in the 200 mg CRS3123 group (129 μg/g) and two (25%) of eight in the 400 mg CRS3123 group (149 μg/g and 503 μg/g) had residual drug in faeces. Patients enrolled under protocol amendment 7 did not have day 17 samples (timepoint was removed).

In the CRS3123 200 mg group, mean plasma CRS3123 concentration was 698 ng/mL (SD 586) 3 h post-dose on day 1 and 437 ng/mL (233) 4 h post-dose at the EOT visit. Plasma CRS3123 concentrations were higher in the CRS3123 400 mg group, with mean values of 1930 ng/mL (SD 1200) 3 h post-dose on day 1 and 1070 ng/mL (692) 4 h post-dose at the EOT visit.

## Discussion

At the TOC visit, patients treated with CRS3123 at either dose showed high rates of clinical cure, similar to those treated with vancomycin. Low recurrence rates compared to vancomycin were observed, particularly at the lower dose of 200 mg twice daily, consistent with the narrow spectrum of activity of CRS3123.^15,16^ CRS3123 at either dose was well tolerated. The most frequently reported adverse events were gastrointestinal, as anticipated in patients with CDI.^25^ There were no clinically significant changes in laboratory evaluations, vital signs, or ECG parameters.

Recurrence of CDI is heavily influenced by ongoing gut dysbiosis. Agents such as metronidazole and vancomycin have a substantial effect on the normal microbiota and thus do not allow for recovery of the microbiota.^26^ This allows for the resurgence or recolonisation of *C difficile* from residual organisms sequestered in the gut that avoided treatment, activation of spores in the gut or from the environment, or reinfection from an environmental pool.^27^ In the previous multiple ascending dose study with CRS3123 in healthy volunteers, the proportion of the major phyla in stool showed minimal changes over time in the 200 mg dosing group and was not different from that in the placebo group.^16^ In the 400 mg and 600 mg twice daily dosing groups, the relative abundance of Bacillota (formerly named Firmicutes) decreased, whereas other phyla, such as Bacteriodota (Bacteroidetes) and Actinomycetota (Actinobacteria), increased. Importantly, no phyla were lost during treatment with CRS3123 at any dose, and the composition was back to baseline levels within 7 days of stopping treatment. At the genus level, no apparent changes were observed after 9 days of twice-daily dosing of CRS3123 in any of the highly abundant intestinal genera except for the target genus *Clostridium*. CRS3123 did not affect commensal anaerobes, including *Bacteroides* and members of *Clostridium* clusters XIVa and IV (eg, *Coprococcus*, *Dorea*, *Roseburia*, and some *Ruminococcus* spp).^16^ These findings are consistent with the narrow spectrum of activity of CRS3123 and align with the observation of a lower recurrence rate for CRS3123 compared with vancomycin in this study. Through study day 40, which is the main timepoint at which recurrence is assessed according to current FDA guidance,^22^ CRS3123 exhibited numerically lower recurrence rates in each of the treatment groups compared with the vancomycin group. Sustained clinical response rates at day 40 were correspondingly higher in the CRS3123 groups than in the vancomycin group.

CRS3123 was generally safe and well tolerated, with only four participants having TEAEs that were possibly related to the study drug, and all were mild to moderate (grade 1 or 2) in severity. The acceptable safety profile of CRS3123 is consistent with low systemic exposure, despite the potential of a leaky gut, which can occur when CDI causes damage to the intestinal lining, potentially altering gut permeability.^28^ In this study of patients with CDI, systemic absorption of CRS3123 remained relatively low, although somewhat higher than that observed in healthy volunteers in our phase 1 studies, consistent with a modest increase in gut permeability in CDI patients.^16,19^

This study, which represents the first clinical testing of CRS3123 in patients with CDI, has some strengths. It was a multicentre study in the USA and Canada and was conducted as part of a collaboration between industry and government. The study had rigorous inclusion criteria, including clinically significant disease manifested by diarrhoea or unformed stools, increased frequency of bowel movements, and verified presence of *C difficile* toxin, thereby ensuring that the patient population had true CDI and not merely *C difficile* colonisation—as is often detected by highly sensitive PCR or glutamate dehydrogenase tests.^29^ Close adherence to the study protocol was evidenced by more than 90% (39/43) of the randomly assigned participants in the ITT population meeting the more restrictive criteria for per-protocol and microbiologically evaluable populations.

Limitations of the study are the small patient numbers due to recruitment challenges during the COVID-19 pandemic, as well as a narrow patient population due to strict exclusion criteria. However, both limitations are consistent with a focused first-in-patient phase 2 study conducted to establish a treatment effect. A specific limitation is that there were few hypervirulent ribotypes among the *C difficile* isolates (three 027 ribotypes and two 078 ribotypes). Both patients with ribotype 078 were assigned to the vancomycin group and had a recurrence; however, it is unknown whether the assigned treatment affected the overall results.

In conclusion, CRS3123 showed high rates of efficacy following 10 days of treatment that were similar to vancomycin. Recurrence rates were numerically lower than those observed with vancomycin and, consequently, sustained clinical response rates at day 40 were higher for patients treated with CRS3123. The safety and efficacy results are promising for this novel, narrow-spectrum agent, and warrant further testing of the drug in phase 3 clinical trials. The final dose selection for phase 3 trials will consider additional omics data (ie, microbiome, metabolome, and inflammation biomarker analysis) and other ancillary study data (ie, developmental and reproductive toxicology and food effects), to be reported separately.

## Supplementary Material

1

## Figures and Tables

**Table 1:** Demographic and baseline characteristics (ITT population)

	CRS3123 200 mg (n=14)	CRS3123 400 mg (n=15)	CRS3123 combined (n=29)	Vancomycin 125 mg (n=14)	Total (n=43)

Age, years					
Mean (SD)	53·0 (18·8)	57·3 (18·8)	55·2 (18·6)	65·0 (15·1)	58·4 (18·0)
Median (IQR)	51·5 (33·8–70·0)	59·0 (42·0–71·0)	58·0 (40·5–70·0)	67·0 (51·3–76·8)	59·0 (43·0–75·0)
Range*	28–81	20–85	20–85	41–90	20–90
Age 18 to <65 years	10 (71%)	10 (67%)	20 (69%)	7 (50%)	27 (63%)
Age ≥65 years	4 (29%)	5 (33%)	9 (31%)	7 (50%)	16 (37%)
Sex					
Female	12 (86%)	11 (73%)	23 (79%)	10 (71%)	33 (77%)
Male	2 (14%)	4 (27%)	6 (21%)	4 (29%)	10 (23%)
Race					
American Indian or Alaska Native	1 (7%)	0	1 (3%)	0	1 (2%)
Asian	2 (14%)	0	2 (7%)	1 (7%)	3 (7%)
Black	0	1 (7%)	1 (3%)	0	1 (2%)
White	11 (79%)	14 (93%)	25 (86%)	13 (93%)	38 (88%)
Ethnicity					
Hispanic or Latinx	1 (7%)	4 (27%)	5 (17%)	8 (57%)	13 (30%)
Not Hispanic or Latinx	13 (93%)	11 (73%)	24 (83%)	6 (43%)	30 (70%)
Height, cm					
Mean (SD)	162·1 (10·8)	162·3 (8·4)	162·2 (9·4)	164·3 (10·7)	162·9 (9·8)
Median (IQR)	163·5 (153·5–170·0)	162·0 (155·0–167·0)	163·0 (155·0–167·5)	162·5 (157·5–171·3)	163·0 (155·0–168·0)
Range*	144–183	150–183	144–183	149–183	144–183
Weight, kg					
Mean (SD)	73·9 (21·3)	74·6 (20·2)	74·2 (20·4)	68·9 (17·3)	72·5 (19·4)
Median (IQR)	71·7 (56·7–83·6)	77·0 (52·7–94·4)	75·0 (55·5–85·4)	66·9 (53·8–84·2)	70·5 (55·0–83·8)
Range*	46–122	43–105	43–122	46–102	43–122
BMI, kg/m^2^					
Mean (SD)	28·0 (6·9)	28·0 (6·3)	28·0 (6·5)	25·4 (5·5)	27·2 (6·3)
Median (IQR)	26·2 (22·6–32·9)	28·8 (21·6–31·1)	27·6 (22·6–31·6)	23·1 (21·5–30·7)	26·6 (21·8–31·1)
Range*	20–42	19–39	19–42	18–36	18–42
C-reactive protein, mg/dL					
Mean (SD)	2·0 (2·6)	2·2 (2·0)	2·1 (2·3)	3·6 (3·8)	2·6 (2·9)
Median (IQR)	0·6 (0·4–3·7)	1·5 (0·4–3·6)	0·8 (0·4–3·6)	1·7 (0·4–7·3)	1·4 (0·4–4·0)
Range*	<0·4–8·2	<0·4–6·8	<0·4–8·2	<0·4–10·6	<0·4–10·6
White blood cell count higher than 15 000 cells per μL	2 (14%)	0	2 (7%)	2 (14%)	4 (9%)
Type of CDI episode					
Primary†	11 (79%)	10 (67%)	21 (72%)	10 (71%)	31 (72%)
First recurrence†	3 (21%)	5 (33%)	8 (28%)	4 (29%)	12 (28%)
Hypervirulent *C difficile* strains					
Ribotype 027	1 (7%)	0	1 (3%)	2 (14%)	3 (10%)
Ribotype 078	0	0	0	2 (14%)	2 (7%)

Data are n (%), unless otherwise stated. Baseline was defined as the last non-missing value recorded before the first dose of study drug. Laboratory data are based on central laboratory data. If the baseline central laboratory value was missing, the local laboratory value was summarised. *C difficile=Clostridioides difficile.* CDI=*C difficile* infection.

* Range shows minimum to maximum values.

† Corrected for one patient in the 400 mg group with a first recurrence who was mis-stratified to the primary episode stratum during randomisation.

**Table 2:** TEAEs reported in the safety population

	CRS3123 200 mg (N=14)	CRS3123 400 mg (N=15)	Vancomycin 125 mg (N=14)	Total (N=43)

Patients with any TEAE	8 (57%)	10 (67%)	10 (71%)	28 (65%)
Gastrointestinal	4 (29%)	4 (27)	6 (43)	14 (33)
Abdominal distension	1 (7%)	0 (0)	1 (7)	2 (5)
Abdominal pain	1 (7%)	0 (0)	3 (21)	4 (9)
Diarrhoea	2 (14%)	1 (7)	4 (29)	7 (16)
Nausea	2 (14%)	1 (7)	0 (0)	3 (7)
Vomiting	2 (14%)	0 (0)	0 (0)	2 (5)
Musculoskeletal and connective tissue	3 (21%)	0 (0)	1 (7)	4 (9)
Muscle spasms	2 (14%)	0 (0)	0 (0)	2 (5)
Nervous system	1 (7%)	4 (27)	0 (0)	5 (12)
Headache	1 (7%)	3 (20)	0 (0)	4 (9)
Skin and subcutaneous tissue	0	3 (20)	0 (0)	3 (7)
Rash	0	2 (13)	0 (0)	2 (5)
TEAEs by maximum severity grade*				
Grade 1	4 (29%)	6 (40%)	4 (29%)	14 (33%)
Grade 2	4 (29%)	4 (27%)	5 (36%)	13 (30%)
Grade 3	0	0	1 (7%)	1 (2%)
Grade 4	0	0	0	0
Grade 5	0	0	0	0
TEAEs by relationship to study drug†				
Not related	7 (50%)	7 (50%)	10 (71%)	24 (56%)
Related or possibly related	1 (7%)	3 (20%)	0	4 (9%)
Serious TEAEs	0	0	1 (7%)	1 (2%)
TEAEs leading to treatment discontinuation	1 (7%)	0	0	1 (2%)

Only TEAEs occurring in two or more patients are shown, leading to some discrepancies in numbers. Verbatim terms were mapped to System Organ Classes and Preferred Terms in MedDRA, version 23.1. TEAE=treatment-emergent adverse event.

* Patients were counted only once, under the highest severity; grades are described in the appendix (p 7).

† Patients were counted only once, under the TEAE with the closest relationship to the study drug.

**Table 3:** Rate of clinical cure at the TOC visit in the ITT, micro-ITT, per-protocol, and microbiologically evaluable populations

	CRS3123 200 mg	CRS3123 400 mg	Vancomycin 125 mg

ITT population	N=14	N=15	N=14
Clinical cure, n (%; 95% CI*)	13 (93%; 66 to 100)	15 (100%; 78 to 100)	13 (93%; 66 to 100)
Clinical failure	0	0	0
Indeterminate response	1 (7%)	0	1 (7%)
Treatment difference (95% CI†)	0 (−26 to 26)	7 (−15 to 32)	Ref
Micro-ITT population	N=13	N=14	N=13
Clinical cure, n (%; 95% CI*)	12 (92%; 64 to 100)	14 (100%; 77 to 100)	13 (100%; 75 to 100)
Clinical failure	0	0	0
Indeterminate response	1 (8%)	0	0
Treatment difference (95% CI†)	−8 (−34 to 17)	0	Ref
Per-protocol and microbiologically evaluable populations‡	N=12	N=14	N=13
Clinical cure, n (%; 95% CI*)	12 (100%; 74 to 100)	14 (100%; 77 to 100)	13 (100%; 75 to 100)
Clinical failure or indeterminate response	0	0	0
Treatment difference†	0	0	Ref

Data are n (%), unless otherwise stated. Clinical cure was defined as survival through the TOC visit and resolution of diarrhoea (ie, less than three UBMs [Bristol Stool Scale score of 5, 6, or 7]) at the EOT visit with maintenance of resolution through the TOC visit and no further requirement (in the investigator's judgement) for treatment for CDI through the TOC visit. The denominator for percentages is the number in the population specified. CDI=*Clostridioides difficile* infection. EOT=end-of-treatment. ITT=intention-to-treat. Micro-ITT=microbiological ITT. TOC=test-of-cure. UBM=unformed bowel movements.

* 95% CIs were calculated using exact Clopper-Pearson confidence intervals.

† Differences (95% CIs) in clinical cure rates between CRS3123 and vancomycin were calculated using the Miettinen and Nurminen method; the 95% CI was not calculated if the number of patients with clinical failure or indeterminate response was zero for both groups.

‡ The per-protocol and microbiologically evaluable populations were identical.

**Table 4:** Rate of sustained clinical response in the micro-ITT, per-protocol, and microbiologically evaluable populations

	CRS3123 200 mg	CRS3123 400 mg	Vancomycin 125 mg

Micro-ITT population	N=13	N=14	N=13
Sustained clinical response			
Yes, n (%; 95% CI*)	12 (92%; 64 to 100)	13 (93%; 66 to 100)	10 (77%; 46 to 95)
No	1 (8%)	1 (7%)	3 (23%)
Treatment difference (95% CI†)	15 (−15 to 45)	16 (−14 to 45)	Ref
Recurrence by day 40 visit‡	0	1 (7%)	3 (23%)
Recurrence by day 70 visit‡	0	2 (14%)	3 (23%)
Per-protocol and microbiologically evaluable populations§	N=12	N=14	N=13
Sustained clinical response			
Yes, n (%; 95% CI*)	12 (100%; 74 to 100)	13 (93%; 66 to 100)	10 (77%; 46 to 95)
No	0	1 (7%)	3 (23%)
Treatment difference (95% CI†)	23 (−5 to 51)	16 (−14 to 45)	Ref
Recurrence by day 40 visit	0	1 (7%)	3 (23%)
Recurrence by day 70 visit	0	2 (14%)	3 (23%)

Data are n (%), unless otherwise stated. Sustained clinical response was defined as clinical cure at the TOC visit without recurrence through the day 40 visit. The denominator for percentages is the number in the population specified. micro-ITT=microbiological intention-to-treat. TOC=test-of-cure.

* 95% CIs were calculated using exact Clopper-Pearson confidence intervals.

† Differences (95% CIs) in sustained clinical response rates between CRS3123 and vancomycin were calculated using the Miettinen and Nurminen method.

‡ Recurrence was assessed in the subset of the micro-ITT population with clinical cure at the TOC visit.

§ The per-protocol and microbiologically evaluable populations were identical.

## Data Availability

In support of data sharing, results are being published, presented at conferences, posted on ClinicalTrials.gov, and shared with the US Food and Drug Administration. Requests for additional data (eg, anonymised individual patient data and supporting clinical documents) should be directed to the stated corresponding authors, UAO (uochsner@crestonepharma.com) and JBB (jbruss@crestonepharma.com), and will be reviewed on a case-by-case basis.

## Research in context

### Evidence before this study

*Clostridioides difficile* infection (CDI) continues to be a global health concern, with about half a million cases and 20 000 to 30 000 deaths annually in the USA alone. Oral vancomycin has been the main treatment since the 1990s but has serious limitations, with recurrence rates of approximately 25%. Metronidazole was previously recommended for treatment of CDI, in part to reduce the selection and propagation of vancomycin-resistant enterococci due to vancomycin use. However, given its broad spectrum of activity, including against other commensal anaerobic bacteria, and disruption of the gut microbiota, metronidazole is now considered a suboptimal therapeutic option. CDI treatment guidelines in the USA were updated in 2018 and again in 2021. The Society for Healthcare Epidemiology of America and Infectious Diseases Society of America currently recommend the more narrow-spectrum agent fidaxomicin over vancomycin, especially to treat recurrent CDI, with faecal microbiota replacement therapy recommended for patients with multiple recurrences.

We performed a search of PubMed on Aug 3, 2025, dating back to Jan 1, 2005, using the terms “difficile” and “Phase 2”, which revealed several studies of antibacterial agents for treatment of CDI, microbiome-restoring modalities to reduce recurrence, and vaccines to prevent CDI. Investigational therapeutics other than CRS3123 and the already approved drug fidaxomicin included surotomycin, cadazolid, ridinilazole, ibezapolstat, LFF571, OPS-2071, MGB-BP-3, and DNV3681. Many of these drug candidates failed to progress to approval for various reasons. Despite the increased use of fidaxomicin, there is a clear unmet medical need for new agents to treat CDI and reduce the rate of recurrence. Key lessons learned from these studies have pointed to the importance of narrow-spectrum agents that preserve a diverse microbiota.

CRS3123 is a methionyl-tRNA ligase inhibitor that targets the type 1 methionyl-tRNA synthetase, also known as methionyl-tRNA ligase type 1 (UniProt Q181D9) found in *C difficile*, enterococci, and in a few gut commensals within the phylum of Bacillota (Firmicutes). CRS3123 is not active against the type 2 methionyl-tRNA synthetase found in many beneficial gut microbes, including Bifidobacteria and other Actinomycetota (Actinobacteria), and most Gram-negative bacteria (eg, Bacteroidota [Bacteroidetes]), which explains its narrow spectrum of activity. Pre-clinical studies showed that CRS3123 exhibits in-vitro activity against clinical isolates of *C difficile* with minimum inhibitory concentrations that range from 0·25 μg/mL to 1·00 μg/mL, including against hypervirulent strains. As a protein synthesis inhibitor, CRS3123 rapidly inhibits *C difficile* toxin production and spore formation. In hamster models, CRS3123 showed superior efficacy to vancomycin, with near 100% survival through the end of the study compared with less than 15% survival for vancomycin. In phase 1 studies, CRS3123 at 200 mg, 400 mg, and 600 mg was well tolerated, showed limited systemic uptake and thus high concentrations in the gut, and caused minimal disruption of normal gut microbiota after 10 days of twice-daily dosing.

### Added value of this study

This phase 2 trial is the first trial of CRS3123 in patients with CDI. The study was conducted as a multicentre, vancomycin-controlled study and applied rigorous inclusion criteria, including a requirement for patients to have toxin-positive diarrhoea, which ensured the patient population had true CDI and not merely *C difficile* colonisation. The phase 2 trial showed not only high rates of clinical cure at the test-of-cure visit in all groups, but also numerically lower rates of recurrence in the CRS3123 dose groups than in the vancomycin group during follow-up, probably due to minimal further disturbance of the gut microbiota by CRS3123. The favourable safety profile of CRS3123—all treatment-emergent adverse events were mild to moderate in this study and similar to vancomycin—is consistent with that noted in two phase 1 studies.

### Implications of all the available evidence

CRS3123 represents a promising narrow-spectrum antibiotic in development for the treatment of CDI. This trial showed safety and efficacy outcomes similar to existing therapies and showed low recurrence rates. Additional evidence from ongoing analyses of CRS3123’s effects on the microbiome, the metabolome, and inflammation biomarkers will help to guide dose selection for progressing CRS3123 into phase 3 clinical trials.
