# Supplementary material for: Safety and efficacy of CRS3123 in adults with a primary episode or first recurrence of Clostridioides difficile infection: a phase 2, randomised, double-blind, multicentre, vancomycin-controlled study
Source: Lancet Infect Dis. Author manuscript; Available in PMC 2026 Feb 27. (PMC12937067; doi:10.1016/S1473-3099(25)00721-2)
Supplement: 1 [file NIHMS2142443-supplement-1.docx]

**SUPPLEMENTARY**

**Safety and efficacy of CRS3123 in adults with a primary episode or first recurrence of *Clostridioides difficile* infection (CDI): A phase 2, randomized, double-blind, vancomycin-controlled study**

Prof. Thomas Louie, MD ^a^, Wendy Ribble, BSc ^b^, Louis Boccumini, MSc ^b^, Katherine Johnson, DO ^b^, Mary A De Groote, MD ^b^, Joshua Day, PhD ^b^, Clifford Mason, PhD ^b^, Xicheng Sun, PhD ^b^, Jane Freeman, PhD ^c^, Kenan Gu, PhD ^d^, Glenn Tillotson, PhD ^e^, Prof. Mark Wilcox, MD ^c^, Nebojsa Janjic, PhD ^b^, Thale Jarvis, PhD ^b^, Seema U Nayak, MD ^d^, Urs A Ochsner, PhD ^b^*, Jon Bruss, MD ^b^*

^a^ Foothills Medical Center, Calgary, AB (Canada)

^b^ Crestone, Boulder, CO (United States)

^c^ University of Leeds - Leeds (United Kingdom)

^d^ National Institute of Allergy and Infectious Diseases, National Institutes of Health, Bethesda, MD (United States)

^e^ GST Micro LLC, North, VA 23128 (United States)

*Authors to whom correspondence should be addressed:

Urs Ochsner, Crestone, Inc., Boulder, CO 80301, USA

[uochsner@crestonepharma.com](mailto:uochsner@crestonepharma.com), Tel. 720-320-6757

Jon Bruss, Crestone, Inc., Boulder, CO 80301, USA

[jbruss@crestonepharma.com](mailto:jbruss@crestonepharma.com), Tel. 269-352-3766

**TABLE OF CONTENTS**

1. Microbiology methods
   1. Specimen requirements
   2. *C. difficile* culture
   3. *C. difficile* spore quantitation
   4. Identification testing
   5. Storage
   6. *C. difficile* semi-quantitative toxin testing
   7. *C. difficile* ribotyping
2. Patient enrollment and disposition
   1. Enrollment challenges
   2. Study populations
   3. Participant disposition
3. Study eligibility criteria
   1. Selection of study population
   2. Exclusion criteria
4. Protocol deviations
5. Risk/benefit assessment
6. References (Supplementary only)
7. **Microbiology methods**
   1. **Specimen requirements**

Fresh, non-preserved stool samples were shipped refrigerated from the Central Laboratory to JMI (now part of Element), North Liberty, IA, weekly via an overnight courier. The patient specimens had to be refrigerated, less than 7 days old prior to freezing, and were tested within one month of frozen.

- 1. ***C. difficile* culture**

All stool specimens were subjected to anaerobic *C. difficile* culture using cycloserine-cefoxitin mannitol broth with taurocholate and lysozyme and cycloserine-cefoxitin-fructose agar for the recovery of *C. difficile*.

- 1. ***C. difficile* spore quantitation**

Spore enumeration was performed on stool specimens after ethanol shock (70% for 60 min) by subculturing serial dilutions onto cycloserine-cefoxitin-fructose agar in duplicate. Spore levels were reported as colony-forming units (CFU) per gram of feces (CFU/g of feces) as described previously (Blanco et al., 2018; Housman et al., 2016).

- 1. **Identification testing**

Identification confirmation of pathogens to genus and species level, as appropriate, was performed using MALDI-TOF MS (Bruker, Germany).

- 1. **Storage**

All *C. difficile* isolates (stored frozen at -80°C) are retained at JMI for three calendar years following completion of the study, with copies to Crestone, Inc. JMI also banked a sweep growth from the *C. difficile* agar, to recover multiple strains of *C. difficile*, if present.

- 1. ***C. difficile* semi-quantitative toxin testing**

Semi-quantitative toxin testing was done at University of Leeds, Healthcare Associated Infection (HCAI) Research Group, U.K., via a validated cell culture cytotoxicity neutralization assay (CCNA). Fecal samples were emulsified in sterile phosphate buffered saline, mixed and centrifuged at 15,000 rpm to remove fecal matter and bacteria. The supernatant was removed and 10-fold dilutions to 10^-6^ were prepared in sterile PBS in sterile 96-well microtiter trays. Equal volumes of the neat sample and TechLab *C. difficile* antitoxin reagent were incubated at room temperature for 30 minutes. A 96-well microtiter tray containing confluent VERO cell monolayers was inoculated with neat sample, neat plus *C. difficile* antitoxin, at dilutions of 10^-1^ to 10^-6^, in duplicate. Assay trays were incubated in a CO_2_ incubator and examined under an inverted microscope at 24h and 48h. The presence of C, difficile toxin in the sample was monitored by >50% cell rounding in the neat sample, with no cell rounding in the corresponding neat plus antitoxin well. The toxin titer was determined by examination of the effect of the dilutions (10^-1^ to 10^-6^) on the VERO cell monolayers. The toxin titer was the reciprocal of the first dilution that showed <50% cell rounding of the VERO cell monolayer.

- 1. ***C. difficile* ribotyping**

PCR ribotyping is the preferred method of genotyping *C. difficile* isolates. It involves amplifying the rDNA of the spacer regions between 16S and 23S ribosomal genes of the organisms. Analysis of the heterogeneity of these regions is the basis for assigning a ribotype. PCR ribotyping of isolates was performed by the *C. difficile* ribotyping Network Reference Laboratory (CDRN)) at University of Leeds, U.K., which is the recognized assigning laboratory for new *C. difficile* PCR ribotypes and has a large reference library of about 1,000 ribotypes.

1. **Patient enrollment and disposition**
   1. Enrollment challenges

The initial target for enrollment was 90-108 patients total, or 30-36 patients per treatment arm. Due to the challenges described below, actual enrollment was lower (N=43), and the study took much longer than expected.

While Phase 2 studies are generally not powered to assess endpoints in the first place, we performed a statistical analysis of the minimum ‘n’ needed per treatment arm to provide an estimate of the true rate of clinical cure at TOC and to provide sufficient comparative safety and pharmacokinetic data in patients with CDI. This analysis indicated that information loss in estimates from as few as 13 subjects per group as compared to 20-36 subjects is minimal with margins-of-error ranging between 0.11 (n=36) and 0.195 (n=13).

Inclusion criteria in our trial were strict, requiring an EIA-positive toxin test. Sites experienced a high rate of screen failures due to negative toxin, anti-CDI medication for >24 hours, ongoing antibiotic therapy, other exclusion criteria (e.g. immunosuppression, IBD), and lack of interest (too many clinic visits). The requirement for a toxin-positive test has also had a highly beneficial consequence of allowing us to only enroll patients who actually have CDI, rather than several other conditions with similar clinical appearance. Therefore, we have a smaller than anticipated, but very high-quality study.

Commonly reported challenges by our clinical trial sites included low incidence of CDI, difficulties to meet the screening window, follow-up visits on weekends, and high prescreen failure rate. Post pandemic there was a site performance "hangover" as staff were reconstituted, and screening and enrollment methods rebuilt which extended the pandemic's negative impact on enrollment.

The impact of the Covid-19 pandemic on enrollment cannot be overstated. Specific Covid effects on study enrollment reported by our investigators ranged from referring physicians’ offices shutting down due to high transmission rates, loss of clinical staff due to illness, referring physicians with limited staff and not taking the time to place patients into a trial, site-specific Covid restrictions acting as a barrier to site activation, patients shying away from healthcare settings, and better infection controls in place resulting in lower frequencies of CDI. Additionally, while our social media campaigns were generally supported by the public, we also received comments of strong opposition to clinical research indicating a significant level of distrust for pharma companies in the Covid era.

Other barriers to enrollment reported by our investigators were reduced testing for CDI in hospitals to avoid financial penalties, and patients not wanting to wait to be treated (i.e., opting for standard-of-care treatment right away rather than being referred to an enrolling investigator).

Initiatives to boost enrollment included brochures, study posters, online search engine optimization (SEO), advertising campaigns (Facebook, LinkedIn, Twitter), physician outreach (Quest, ‘Dear Doctor’ letters), Virtual Waiting Room (SubjectWell), newsletters to sites, presentations at patient advocacy meetings (e.g. Peggy Lillis Foundation), radio and billboard ads, and regular study coordinator huddles. We also amended the protocol to remove Day 4 and D17 in-person visits and to relax some of the exclusion criteria around HIV and immunodeficiency.

There is a recent precedent: Ibezapolstat (AcuRx) had 40 patients in their combined Phase 2a/2b CDI trial (NCT04247542) and had a successful outcome of an End-of-Phase 2 meeting with the FDA, supporting the advancement into Phase 3 studies (Eubank et al., 2025). Also, the fidaxomicin (OPT-80) trial had 48 participants and three arms (Louie et al., 2009), so there is a history of smaller Phase 2 trials, including for the only new CDI drug, fidaxomicin, that eventually got approval.

- 1. Study populations

A schematic of study populations and definitions is provided in Figure S1.

**Figure S1: Study Populations.**

Abbreviations: EOT = end of treatment; ITT = intent-to-treat; ME = microbiologically evaluable; micro-ITT = microbiological intent-to-treat; PK = pharmacokinetic; PP = Per Protocol; MRT= microbiota restoration therapy.


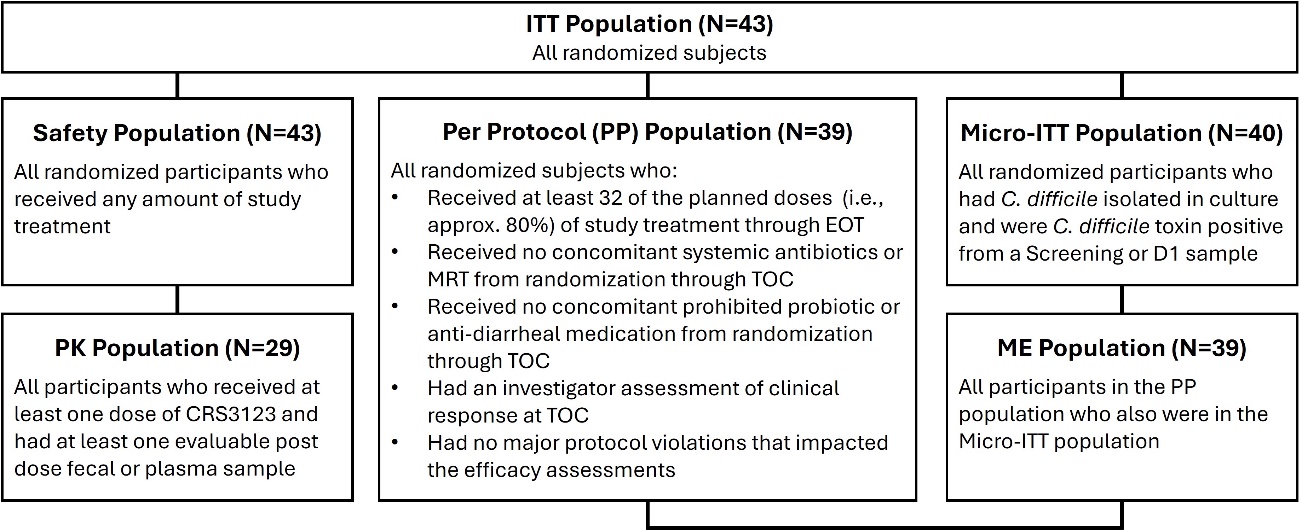


- 1. Participant disposition

Details of participant disposition are provided for the ITT population in Table S1.

Forty-three participants were randomized into the study, including 14 participants in each of the CRS3123 200 mg bid and vancomycin 125 mg qid groups, and 15 participants in the CRS3123 400 mg bid group.

Forty-two (42/43) participants in the ITT population completed study treatment. One participant (in the CRS3123 200 mg bid group) did not complete treatment withdrawing on Day 9 of the study. Overall, forty (40/43) participants in the ITT population completed the study. Three participants including the above-mentioned patient did not complete the study, including one in the vancomycin 125 mg group (Day 11, prior to TOC and another in the CRS3123 200mg group who completed the Day 40 visit and deemed lost to follow-up after several contact attempts.

**Table S1 Participant Disposition (ITT Population)**


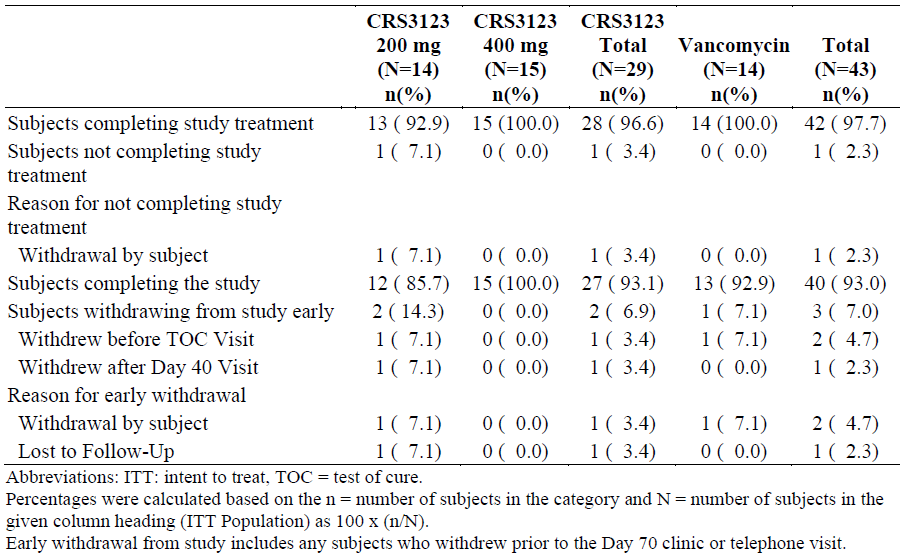


1. **Study eligibility criteria**
   1. **Selection of study population**

In order to be considered eligible for the Phase 2 study, all of the following criteria must have been met:

*Age*

1. Adults, ≥ 18 years of age

*Type of Participant and Disease Characteristics*

1. More than or equal to 3 diarrheal (Bristol Stool Scale scores 5, 6, or 7) stools/day in a 24-hour period during screening prior to randomization and in the judgment of the investigator that C. difficile was the likely causative agent for the diarrhea.
2. Stool positive for C. difficile Toxin A and/or B antigen using an FDA or Health Canada approved/cleared EIA or ELISA laboratory test (e.g., Abbott/Alere QUIK CHEK COMPLETE^®^, Premier^®^ Toxins A&B, etc.). If the QUIK CHEK COMPLETE® test was completed in a laboratory that was not CLIA-certified, the test had to be performed according to the package insert, results verified by a second individual and a photograph of the result recorded with the date and time of the result.
3. Participants with a primary episode or first recurrence of CDI are eligible.
4. In the judgment of the investigator, the expectation that the participant would survive with effective antibiotic therapy and appropriate supportive care for the anticipated duration of the study.

*Sex*

1. Female participants of childbearing potential could not be pregnant (have a negative urine pregnancy test [β-hCG] at screening), plan to become pregnant during the study, or be breastfeeding; and had to be willing to commit to either sexual abstinence or use highly effective methods of birth control contraception from screening through FUV3. Note: Female participants of non‑childbearing potential are defined as those that are either postmenopausal as demonstrated by amenorrhea for ≥12 months, or who have had surgical sterilization (i.e., tubal ligation, bilateral salpingectomy, bilateral oophorectomy, or hysterectomy). Provision of documentation was not required for female sterilization; verbal confirmation was sufficient.
2. Males had to use a condom and spermicide from screening through FUV3 if the female partner(s) was of childbearing potential and could not donate sperm from screening through FUV3. This criterion also applied to males who were surgically sterile.

*Informed Consent*

1. Capable of giving signed informed consent, which includes compliance with the requirements and restrictions listed in the informed consent form and in this protocol.
   1. **Exclusion criteria**

Participants who met any one of the following criteria were ineligible for participation in the study:

*Medical Conditions*

1. Participants with any of the following conditions:
2. Intractable vomiting preventing oral medication intake.
3. Severe underlying disease with an expected survival time less than the duration of the study (approximately 70 days).
4. More than 1 prior CDI occurrence within the last 3 months or more than 2 prior episodes of CDI in the last 12 months.
5. A history of a recent CDI episode within 3 months prior to enrollment that was non‑responsive to vancomycin.
6. In the investigator’s opinion, the participant was anticipated to require oral or intravenous systemic antibiotic therapy for a non-CDI infection between screening and FUV3.
7. Inflammatory bowel disease (Crohn’s disease or ulcerative colitis), uncorrected Hirschsprung’s disease, short gut syndrome, or any other condition known to significantly impact bowel motility and/or malabsorption.
8. Any other known pathogen associated with diarrhea.
9. Life-threatening or fulminant CDI as defined by IDSA/SHEA Guidelines ([McDonald et al, 2018](#McDonald_2018)):
10. Any signs of severe sepsis, including shock or profound hypotension
11. Ileus
12. Toxic megacolon
13. Colonic perforation
14. Need for concurrent laxatives or tube feeds, toxin binders, bile acid sequestrants during the study. Microbiota restoration therapy (MRT) or any phage therapy within 1 year of randomization. Receipt of bezlotoxumab within 3 months of randomization.
15. Participants treated with another antimicrobial agent directed at the current episode of CDI (metronidazole, fidaxomicin, rifaximin, tigecycline, or oral vancomycin) for >24 hours (e.g., four doses of vancomycin within 24 hours) of treatment within the 3 days prior to randomization were not eligible for enrollment.
16. Pregnant or breastfeeding women
17. Receipt of any investigational medication during the last month (30 days or 5 half-lives, whichever is longer) prior to randomization
18. Active and uncontrolled HIV with CD4 <200/mm^3^
19. Presence of active malignancy undergoing chemotherapy that was expected to cause significant immunosuppression, hematologic malignancy undergoing induction chemotherapy, or recent bone marrow or solid organ transplant (within 1 month prior to randomization) undergoing treatment with medications for the rejection of transplantation. In the investigator's opinion, was expected not to survive through the duration of the study (approximately 70 days) due to complications of the malignancy, or in the investigator’s opinion would require oral or intravenous systemic antibiotic therapy during the study for malignancy-related conditions.
20. Severe neutropenia defined as ANC <500 cells/mm^3^
21. Severe hepatic impairment at screening including clinical signs of cirrhosis, end-stage hepatic disease (e.g., ascites, hepatic encephalopathy), or alanine aminotransferase (ALT) or aspartate aminotransferase (AST) ≥3x upper limit of normal (ULN) or total bilirubin ≥2x ULN
22. Any other surgical or medical condition (including a clinically significant laboratory abnormality) as determined by the investigator or the medical monitor, that could interfere with the participant’s ability to participate in the study, the administration of study treatment, and/or the interpretation of study results that, in the investigator’s opinion, could confound study assessments or study procedures
23. Known hypersensitivity to CRS3123 or oral vancomycin
24. An employee of the investigator or study center with direct involvement in the proposed study or other studies under the direction of that investigator or study center, as well as a family member of the employee or the investigator
25. Unwillingness to stop consuming non-dietary probiotics from randomization to FUV3. (Non‑dietary probiotics include capsules, powders, or liquids that are nutraceutical probiotics [primary ingredient is bacteria or yeast]. Yogurt, kombucha, kimchi, kefir, brine pickles, cheese, and other foods that are considered “dietary probiotics” were permitted.)
26. Participants currently taking digoxin within 1 week of screening
27. Unwillingness to refrain from consumption of grapefruit and its juices as well as nutraceutical supplements containing curcumin (i.e., turmeric) from randomization until 24 hours after EOT
28. Unwillingness to stop use of antidiarrheals from randomization to FUV3

*Prior/Concurrent Clinical Study Experience*

1. Previous enrollment in this study or previous treatment with CRS3123
2. **Protocol Deviations**

The majority (38/43; 88%) of the participants in the Safety population exhibited at least one protocol deviation, most of which were minor deviations.

There were 5 major protocol deviations:

• One in the CRS3123 200 mg group received prohibited concomitant medication (participant re-started cholestyramine for bile salt diarrhea [post cholecystectomy]) at TOC (Day 12).

• One in the CRS3123 200 mg group failed to meet inclusion criterion #3 at screening (the QCC test was negative for Toxin, the Cepheid test detected Toxin B gene target.

• One in the CRS3123 400 mg group failed to meet inclusion criterion #3 at screening. At the time of testing only the GDH was positive, and the Toxin A and B were negative.

• One in the CRS3123 400 mg group had a TOC/FUV1 visit on Day 15 which was done out of window due to PI being out of office.

• One in the vancomycin 125 mg group received an infusion of Rituxan for myasthenia gravis by a non-study neurologist prior to screening but this had not been reported.

1. **Risk/benefit Assessment**

A risk/benefit section was included in the IND/IB and described in the ICF. For each visit during the trial, risks were systematically assessed using the CTCAE v5 grading scale (below) as per SAP and study protocol, with statistical analysis of AEs and changes in laboratory values to determine incidence, severity and intensity of events. There was regular DSMB oversight.

Grade refers to the severity of the AE. The CTCAE displays Grades 1 through 5 with unique clinical descriptions of severity for each AE based on this general guideline:

- Grade 1 Mild; asymptomatic or mild symptoms; clinical or diagnostic observations only; intervention not indicated.
- Grade 2 Moderate; minimal, local or noninvasive intervention indicated; limiting age-appropriate instrumental ADL*.
- Grade 3 Severe or medically significant but not immediately life-threatening; hospitalization or prolongation of hospitalization indicated; disabling; limiting self care ADL**.
- Grade 4 Life-threatening consequences; urgent intervention indicated.
- Grade 5 Death related to AE.

1. **References (Supplementary Section only)**

Blanco N, Walk S, Malani AN, Rickard A, Benn M, Eisenberg M, Zhang M, Foxman B. *Clostridium difficile* shows no trade-off between toxin and spore production within the human host. *J Med Microbiol* 2018; **67:** 631-40.

Housman ST, Banevicius MA, Lamb LM, Nicolau DP. Isolation and quantitation of *Clostridium difficile* in aqueous and fecal matter using two types of selective media. *J Microbiol Immunol Infect* 2016; **49:** 445-7).

Eubank TA, Jo J, Alam MJ, Begum K, McPherson JK, Le TM, et al. Efficacy, safety, pharmacokinetics, and associated microbiome changes of ibezapolstat compared with vancomycin in adults with *Clostridioides difficile* infection: a phase 2b, randomised, double-blind, active-controlled, multicentre study. *Lancet Microbe* 2025; **6:** 101126.

Louie T, Miller M, Donskey C, Mullane K, Goldstein EJ. Clinical outcomes, safety, and pharmacokinetics of OPT-80 in a phase 2 trial with patients with *Clostridium difficile* infection. *Antimicrob Agents Chemother* 2009; **53:** 223-8.

McDonald LC, Gerding DN, Johnson S, Bakken JS, Carroll KC, Coffin SE, Dubberke ER, Garey KW, Gould CV, Kelly C, Loo V, Sammons JS, Sandora TJ, Wilcox MH. Clinical Practice Guidelines for *Clostridium difficile* Infection in Adults and Children: 2017 Update by the Infectious Diseases Society of America (IDSA) and Society for Healthcare Epidemiology of America (SHEA). Clin Infect Dis 2018; **66:** e1-e48.
